# Validation of the Cornell Musculoskeletal Discomfort Questionnaires in textile workers in Peru

**DOI:** 10.47626/1679-4435-2023-1029

**Published:** 2024-02-16

**Authors:** Jonh Maximiliano Astete-Cornejo, Jerusca Rubi Asencios-Hidalgo

**Affiliations:** 1 Universidad Peruana Cayetano Heredia, Unidad de Medicina Ocupacional y Medio Ambiente, Lima, Perú; 2 Instituto Nacional de Salud, Centro Nacional de Salud Ocupacional y Protección del Ambiente para la Salud, Lima, Perú

**Keywords:** musculoskeletal system, occupational medicine, occupational health, ergonomics, sistema musculoesquelético, medicina del trabajo, salud laboral, ergonomía

## Abstract

**Introduction:**

Musculoskeletal complaints are considered occupational health problems in developing countries with manufacturing companies. In our country there are no validated questionnaires that can be used to screen for musculoskeletal complaints.

**Objectives:**

To adapt and validate the Cornell Musculoskeletal Discomfort Questionnaires instrument into Spanish. A cultural adaptation and validation of the questionnaire was carried out in the following stages: translation, expert review, retranslation, and validation of the adapted version. The study was conducted with workers from a textile company in Lima, Peru.

**Methods:**

Content validation was carried with 10 experts; criterion validity, with 42 workers; discriminant validity and internal consistency, with 35 textile workers and 35 non-textile; and, finally, test-retest reliability, with 30 workers.

**Results:**

The results obtained showed content validity, with the value of Aiken’s V for each question being greater than 0.7 for clarity, relevance, and sufficiency, and criterion validation obtained correlation values greater than 0.7 in most items. Moreover, sensitivity 97.1% for body parts, 17.1% for right hand, and 28.6% for left hand, whereas specificity was 0% for body parts, 14.3% for left hand, and 28.6% for right hand. Finally, test-retest Spearman’s correlation was 0.69.

**Conclusions:**

The study allows us to conclude that the Cornell Musculoskeletal Discomfort Questionnaire-JAH is a valid and reliable instrument for the exploration of musculoskeletal discomfort amongst workers in the textile sector.

## Abstract (es)

**Introducción:**

Las molestias musculoesqueléticas son consideradas problemas de salud laboral en países con empresas manufactureras y en vías de desarrollo, y en Perú no se cuenta con cuestionarios validados que puedan utilizarse para tamizar molestias musculoesqueléticas.

**Objetivos:**

Adaptar y validar al español el instrumento Cornell Musculoskeletal Discomfort Questionnaires. Se realizó una adaptación cultural y validación de cuestionario en etapas: traducción, revisión de expertos, retraducción y validación de la versión adaptada. El estudio se realizó con trabajadores de una empresa textil de Lima, Perú.

**Métodos:**

Se realizó la validación de contenido con 10 expertos; la validez de criterio, con 42 trabajadores; la validez discriminante y la consistencia interna, con 35 trabajadores textiles y 35 trabajadores que no pertenecían al sector; y, por último, la fabilidad test-retest, con 30 trabajadores.

**Resultados:**

Los resultados obtenidos mostraron validez de contenido, con el valor de V de Aiken para cada pregunta presentando valores superiores a 0,7 tanto para claridad, pertinencia y suficiencia, y la validez de criterio presentó valores de correlación superiores a 0,7 en la mayoría de los ítems. La sensibilidad fue del 97,1% para partes del cuerpo, 17,1% para mano derecha, y 28,6% para mano izquierda, mientras la especificidad fue del 0% para partes de cuerpo 14,3% para mano izquierda, y 28,6% para mano derecha. Finalmente, hubo una correlación de Spearman en el test-retest de 0,69.

**Conclusiones:**

El estudio permite concluir que el instrumento Cornell Musculoskeletal Discomfort Questionnaire-JAH es válido y fiable para la exploración de molestias musculoesqueléticas para aplicarlo en trabajadores del sector textil.

## INTRODUCTION

Musculoskeletal diseases are conditions that mainly affect muscles, tendons, and nerves. These injuries are considered a public health problem when associated with the work activities performed by the person with the injury. The costs arising from these conditions represent an important problem in developing countries such as Peru, thus having an impact on workers’ productivity and well-being.^1^

It is known that the textile sector in Peru has developed taking advantage of the country’s ecological assets. Throughout history, craft workshops and subsequently industrial plants have been developed, leading to the need for workforce and scientific knowledge on processes of different complexity and resulting in exposure of many people to several risks inherent to the activity they perform. Currently, according to a study prepared by the Peruvian Ministry of Foreign Trade and Tourism, the economic recovery observed in the main countries that demand Peru’s products will bring additional benefits to the textile and clothing industry yearly, and thus generate a higher demand for workforce, with the consequent exposure to risk inherent to their work, in addition to increasing their risk to develop musculoskeletal diseases.^2,3^

The Health and Labor Institute (Instituto Salud y Trabajo, SAT), considers disergonomic risk to be the first risk factor to which workers are exposed (96.4%), followed by psychosocial risk (48.3%), noise (34.9%), biological risk (28.6%), and dust (19,8%). The exposed population is distributed into the several economic activities, the most important of which are: public administration, food product preparation, textile manufacturing, manufacturing of industrial chemical substances, and manufacturing of other types of transport equipment.^4^

The National Health Institute, through the National Center for Occupational Health and Environmental Health, conducted an investigation to understand the working, safety and health conditions of the urban economically active and employed population in Peru. With regard to exposure to occupational risk factors, it was found that workers performed tasks that made them keep uncomfortable or forced postures (12.9%) or to make repetitive movements (21.6%).^5^

Taking into account the morbidity of the economically active population (EAP) in Peru, according to the general profile of the population covered by the Peru Health Insurance (Seguro Social de Salud, EsSalud), musculoskeletal diseases account for 15.8% of overall morbidity. This group includes back pain (5.4%, with a higher percentage in individuals aged from 30 to 64 years), followed by arthrosis (3.2%, with higher frequency in people aged 45 years or older).^4^

Since disergonomic risks are among the most common ones to which the EAP are exposed, it is important to develop health screening instruments to be used in clinical practice and research, to timely evaluate musculoskeletal symptoms in this working population. Therefore, it is proposed to culturally adapt a screening instrument for musculoskeletal diseases and confirm its psychometric characteristics, such as reliability, validity, sensitivity, and feasibility.

The Cornell Musculoskeletal Discomfort Questionnaires (CMDQ) evaluates musculoskeletal symptoms in sedentary workers and standing workers, as well as hand symptoms.^6,7^ There is information confirming the utility of CMDQ to measure musculoskeletal diseases produced in the workplace.^8,9,10^

The present study aims to determine content, criterion and discriminant validity, internal consistency, and test-retest reliability of the CMDQ instrument.

## METHODS

This is a cross-sectional questionnaire validation study with data collected from a textile company in the city of Lima, Peru.

Sampling was random, the sample consisted of the list of workers at the textile company, and the following sample sizes were obtained, according to different types of validation, with a 95% confidence level: 10 experts for content validation, 35 textile workers and 35 non-textile workers for internal consistency and discriminant validity, and 42 textile workers for criterion validation and 30 workers for test-retest reliability, who answered the CMDQ instrument to evaluate musculoskeletal disorders.

The CMDQ questionnaire is a screening tool but not a diagnostic instrument and includes six questionnaires, which assess musculoskeletal symptoms (sedentary workers, standing worker, and hand symptoms) both for men and for women. These questionnaires are based on previous studies on musculoskeletal diseases among office workers. The scores of the questionnaires should be evident for any person familiarized with this type of investigation.^7,11^

The six components of the original CMDQ instrument were translated by a certified American translator; subsequently, the translated questionnaires were reviewed by physicians specialized in occupational medicine and ergonomics and experienced in the management of musculoskeletal disorders; finally, the translated questionnaires translated into Spanish were retranslated into English by two translators: an American who had lived in Peru for more than 5 years and a Peruvian translator who had lived in the USA for more than 5 years. None of the two translators was familiar with the original document in English.^12,13,14^

## RESULTS

Content validity was determined through the evaluation by 10 judges, who assessed clarity, relevance, and sufficiency of the questionnaire questions with scores ranging from 0 to 10.

Aiken’s V values were obtained for each question, and all items had values greater than 0.7 for clarity, relevance, and sufficiency, and most items had a 95% confidence interval lower limit above 0.5. The lowest value was found for sufficiency of question 1, which obtained a value of 0.5 (Table 1).

**Table 1:** Aiken V values for content validity

Questions	Validity	Aiken’s V	Limits	95%CI
Question 1: During the last work week, how often did you experience pain, ache, and/or discomfort?	Clarity	0.8	Lower	0.63
a. Never			Upper	0.88
b. 1-2 times last week	Relevance	0,7	Lower	0.52
c. 3-4 times last week			Upper	0.8
d. Once every day	Sufficiency	0,7	Lower	0.5
e. Several times every day.			Upper	0.78

Question 2: If you experienced ache, pain, and/or discomfort, how uncomfortable was this?	Clarity	0,8	Lower	0.65
a. Slightly uncomfortable			Upper	0.9
b. Moderately uncomfortable	Relevance	0,7	Lower	0.5
c. Very uncomfortable			Upper	0.78
	Sufficiency	0,7	Lower	0.52
			Upper	0.8

Question 3: If you experienced ache and/or discomfort, did this affect your ability to work?	Clarity	0,9	Lower	0.74
a. Not at all			Upper	0.95
b. Slightly	Relevance	0,7	Lower	0.52
c. Substantially			Upper	0.8
	Sufficiency	0,8	Lower	0.63
			Upper	0.88

The correlation between results for the CMDQ and the visual analogue scale (VAS) was found to be greater than 0.7 in most items, indicating a high correlation, except for the following items: right and left upper arm, hip/buttocks, right and left thigh, right and left knee, right and left foot, right and left hand (regions A, B, C, D, E, F) (Table 2).

**Table 2:** Criterion validity of the Cornell Musculoskeletal Discomfort Questionnaires questionnaire

Body parts	Kappa	Spearman
Neck	1.000	0.997
Right shoulder	0.919	0.924
Left shoulder	0.806	0.830
Upper back	0.604	0.624
Right upper arm	0.540	0.591
Left upper arm	0.481	0.577
Lower back	0.857	0.922
Right forearm	0.656	0.698
Left forearm	1.000	1.000
Right wrist	1.000	0.999
Left wrist	1.000	1.000
Hip/buttocks	0.644	0.689
Right thigh	0.624	0.624
Left tight	0.624	0.624
Right knee	0.624	0.624
Left knee	0.624	0.624
Right lower leg	1.000	1.000
Left lower leg	1.000	1.000
Right foot	0.644	0.689
Left foot	0.644	0.689
Right hand A	0.189	0.212
Right hand B	0.624	0.624
Right hand C	−0.044	−0.075
Right hand D	0.624	0.624
Right hand E	0.488	0.459
Right hand F	0.236	0.245
Left hand A	-0.073	-0.082
Left hand B	0.624	0.624
Left hand C	0.656	0.698
Left hand D	0.624	0.624
Left hand E	0.364	0.362
Left hand F	0.288	0.299

Discriminant validity was analyzed by comparing the results of a survey with 35 textile workers (exposed) and 35 non-textile workers (unexposed). The sensitivity was 97.1% for body parts, 28.6% for left hand, and 17.1% for right hand, whereas specificity was 0% for body parts, 14.3% for left hand, and 28,6% for right hand (Table 3).

**Table 3:** Discriminant validity of the Cornell Musculoskeletal Discomfort Questionnaires questionnaire

	Sensitivity	Specificity
Body parts	97.1%	0.0%
Left hand	28.6%	14.3%
Right hand	17.1 %	28.6%

The analysis of CMDQ instrument reliability through an internal consistency test obtained a Cronbach’s alpha of 0.84 for body parts (mean), a Cronbach’s alpha of 0.93, 0.87, 0.96, 0.86, 0.96, 0.96, 0.82, 0.96, 0.80, 0.97, 0.88 for neck, shoulders, upper back, upper arms, lower back, forearms, wrists, hip, thighs, knees, and feet, respectively, and a total Cronbach’s alpha of 0.91, as shown in Table 4.

**Table 4:** Internal consistency of the Cornell Musculoskeletal Discomfort Questionnaires questionnaire

	Cronbach’s alpha
Body parts	0.84
Neck	0.93
Shoulders	0.87
Upper back	0.96
Upper arms	0.86
Lower back	0.96
Forearms	0.96
Wrists	0.82
Hip	0.96
Thighs	0.80
Knees	0.97
Lower legs	0.99
Feet	0.88
Left hand	0.84
Right hand	0.90
Total	0.91

For the assessment of test-retest reliability, the CMDQ questionnaire was administered for a second time to a group of 35 people, at an interval from 5 to 7 days after the first administration. The Shapiro-Wilk test for the two administrations yielded values <0.05 in most items; therefore, it was decided to use the classical Spearman’s correlation coefficient (Table 5).

**Table 5:** Test-retest reliability of the Cornell Musculoskeletal Discomfort Questionnaires questionnaire

	Shapiro-Wilk		
	Correlation coeficient	Degrees of freedom	Significance
Total first administration	0.820	30	0.000
Total second administration	0.931	30	0.052
Body parts first administration	0.891	30	0.005
Body parts second administration	0.923	30	0.031
R hand first administration	0.516	30	0.000
R hand second administration	0.473	30	0.000
L hand first administration	0.358	30	0.000
L hand second administration	0.275	30	0.000

L = left; R = right.

Total test-retest Spearman’s correlation was 0.691 (significant), as observed in Table 6, whereas it was 0.682 (significant) for body parts, 0.906 (significant) for right hand, and 0.356 for left hand, a value that revealed a low (non-significant) correlation, considering a significance level higher than 0.05.

**Table 6:** Test-retest reliability with Spearman’s correlation

	Rho coefficient	Significance
Total	0.691	0.000
Body parts	0.682	0.000
Right hand	0.906	0.000
Left hand	0.356	0.054

## DISCUSSION

Content validity showed that all questions of the questionnaire were clear, understandable, and coherent. Aiken’s V values were greater than 0.7, and most had a 95% confidence interval lower limit above 0.5, which makes it possible to ensure this result. Previous validation studies of this questionnaire did not perform this type of validation; therefore, comparative data are not available.

With regard to criterion validity, answers for VAS and CMDQ were analyzed using the Cohen’s kappa coefficient and the Spearman’s correlation coefficient. Kappa values allowed for interpreting the agreement between CMDQ and VAS, with values closer to 1 indicating a higher level of agreement.

The results of the present study showed that agreement was high, since kappa values were mostly greater than 0.7, as observed in most body parts evaluated, such as neck, left forearm, right and left wrist, among others, which means that both CMDQ and VAS measure the presence or absence of musculoskeletal symptoms. The study to validate the Turkish version of the questionnaire (T-CMDQ) found similar results, with kappa ranging from 0.61 to 0.91 across body parts, which indicated a substantial or almost perfect agreement between VAS and T-CMDQ scores. In the study to validate the Equatorian version of the questionnaire (E-CMDQ), kappa values ranged from 0.6 to 0.9 across body parts, indicating a good agreement between VAS and E-CMDQ scores.^15^

An analysis of the correlation between CMDQ and VAS scores in the present study revealed that correlation exists in most body parts, such as neck, right shoulder, left forearm. In the Turkish validation study, Spearman’s correlation coefficients ranged from 0.46 to 0.83 across body parts, indicating that VAS and T-CMDQ scores were positively correlated.^16^

Concerning discriminant validity in this study, the sensitivity of the questionnaire was 97.1% to evaluate body parts, which was interpreted as high sensitivity. Conversely, the sensitivity for both right and left hands presented values as low as 17.1% and 28.6% respectively, showing that the questionnaire is not sensitive to identify hand symptoms. In the previous validations of other versions of the questionnaire, such as Turkish, German, and Equatorian ones, this aspect was not considered nor evaluated.

In relation to specificity, the present study found levels as low as 0%, 14.3%, and 28.6% for body parts, left hand, and right hand respectively, which means that the CMDQ is not able to properly distinguish exposed and unexposed populations. This result may be explained by the fact that non-textile workers are exposed to risk other than the ergonomic one, and they are likely to also present some type of work-related musculoskeletal discomfort. Therefore, the instrument was not able to distinguish which ailments were specific of the textile sector, since it identified discomfort in general without distinguishing any type of ailment. Due to this finding, it is recommended to conduct additional studies with greater specification and other risk groups.

With regard to internal consistency, Cronbach’s alpha for the 96 items was 0.91, which means that the correlation between the items was excellent; thus, reliability was excellent. This was also evidenced in the Cronbach’s alpha for body parts, right hand, and left hand. The validation of the German version of the CMDQ questionnaire (D-CMDQ) obtained a Cronbach’s alpha of 0.82, which was considered a very adequate value,^17^ whereas the E-CMDQ had a Cronbach’s alpha of 0.8, indicating that its internal consistency was high.^15^

As for test-retest reliability, the CMDQ questionnaire was administered for a second time to a group of 35 people, obtaining results in agreement with the validation of the D-CMDQ, in which test-retest scores ranged from 0.56 to 0,72, showing a moderate, highly significant correlation.^17^

## CONCLUSIONS

The CMDQ instrument validated in the Universidad Peruana Cayetano Heredia is valid and reliable, showing content and criterion validity, as well as high test-retest reliability, with results maintained over time.

The instrument is sensible to identify musculoskeletal diseases affecting the body.
